# Ameliorating effects and mechanisms of transcutaneous auricular vagal nerve stimulation on abdominal pain and constipation

**DOI:** 10.1172/jci.insight.150052

**Published:** 2021-07-22

**Authors:** Xiaodan Shi, Yedong Hu, Bo Zhang, Wenna Li, Jiande DZ Chen, Fei Liu

**Affiliations:** 1 Department of Gastroenterology, Shanghai East Hospital affiliated to Tongji University, Shanghai, China.; 2 Department of Gastroenterology, the 928th Hospital of the PLA Joint Logistics Support Force, Haikou, Hainan, China.; 3 Division of Gastroenterology and Hepatology, University of Michigan, Ann Arbor, Michigan, USA.

**Keywords:** Gastroenterology, Pain

## Abstract

**Background:**

Abdominal pain and constipation are 2 main symptoms in patients with constipation-predominant irritable bowel syndrome (IBS-C). This study aimed to investigate the effects and possible mechanisms of transcutaneous auricular vagal nerve stimulation (taVNS) in patients with IBS-C.

**Methods:**

Forty-two patients with IBS-C were randomized into a 4-week sham-taVNS or taVNS treatment. The primary outcomes were complete spontaneous bowel movements per week (CSBMs/week) and visual analog scale (VAS) for abdominal pain. High-resolution anorectal manometry (HRAM) was performed to evaluate anorectal motor and sensory function. Cytokines and brain gut peptides were analyzed in blood samples. ECG was recorded for the assessment of autonomic function.

**Results:**

Compared with sham-taVNS, (a) taVNS increased CSBMs/week (*P* = 0.001) and decreased VAS pain score (*P* = 0.001); (b) improved quality of life (*P* = 0.020) and decreased IBS symptom score (*P* = 0.001); (c) improved rectoanal inhibitory reflex (*P* = 0.014) and improved rectal sensation (*P <* 0.04); (d) decreased a number of proinflammatory cytokines and serotonin in circulation; and (e) enhanced vagal activity (*P* = 0.040). The vagal activity was weakly correlated with the CSBMs/week (*r* = 0.391; *P* = 0.010) and the VAS pain score (*r* = –0.347; *P* = 0.025).

**Conclusions:**

Noninvasive taVNS improves both constipation and abdominal pain in patients with IBS-C. The improvement in IBS-C symptoms might be attributed to the integrative effects of taVNS on intestinal functions mediated via the autoimmune mechanisms.

**Trial registration:**

www.chictr.org.cn, no. ChiCTR2000029644.

**Funding:**

National Natural Science Foundation of China (grant no. 81970538 for FL).

## Introduction

Irritable Bowel Syndrome (IBS) is a common functional gastrointestinal disorder characterized by abdominal pain and altered bowel habits in the absence of demonstrable organic disease (1, 2). IBS affects 7%–21% of individuals globally (3, 4), with constipation-predominant IBS (IBS-C) accounting for approximately one-third of cases (5, 6). The syndrome is non–life-threatening, but it has a significant personal and social impact, resulting in a decline of the quality of life and an increase of medical burden (7, 8).

The etiology and pathogenesis of IBS have not been fully elucidated, but many factors are involved, including genetic factors, intestinal infection, mucosal immune and inflammatory response, altered gastrointestinal motility, visceral hypersensitivity, postinfectious reactivity, brain-gut interactions, disturbance of intestinal flora, food sensitivity, or stress (9–11), among which altered gastrointestinal motility and visceral hypersensitivity have been considered to be the main pathophysiological basis (12, 13). At present, the treatment of IBS typically addresses the predominant symptom experienced by the patient. Constipation and abdominal pain are the main complaints of IBS-C, which are difficult to treat at the same time. The current management for IBS-C includes lifestyle modification, specialized diets, psychological treatment, and pharmacologic therapies (1). Various drugs are used for pharmacologic treatment. Laxatives and prokinetics are used for constipation, while antispasmodics and antidepressants are prescribed for abdominal pain, which in turn may impair gastrointestinal motility and, thus, could worsen constipation (14, 15). Additionally, such medications can only relieve symptoms temporarily, and long-term medication is expensive and easy to relapse after withdrawal (16). Accordingly, patients often seek complementary and alternative therapies (17).

The autonomic nervous system, composed of the sympathetic and parasympathetic nerves (18), is known to play an important role in the brain-gut control of the gastrointestinal functions (19, 20). Accordingly, vagus nerve stimulation (VNS) is of interest as a potential therapeutic intervention. VNS, approved by the US Food and Drug Administration (FDA) for epilepsy and pharmaco-resistant depression (21), has also been explored for its therapeutic potentials for gastrointestinal dysmotility, inflammation, and pain (22–24). The implantable device for VNS consists of an electrode that is wrapped around the left vagus nerve, and an implantable pulse generator positioned below the collarbone. Due to the involvement of surgery, perioperative risks, and potential side effects, the invasvie VNS has not been approved for treating any diseases of the gut.

Transcutaneous VNS (tVNS) is a noninvasive method that has been developed to overcome the limitations of the invasive VNS. The rationale for using tVNS on the ear is based on anatomical studies that suggest that the ear is the only place on the surface of the human body where there is afferent vagus nerve distribution (25, 26). Thus, noninvasive stimulation of the afferent nerve fibers on the ear should produce effects similar to invasive VNS. In recent years, transcutaneous auricular VNS (taVNS) has been reported to improve gastrointestinal disorders. A recent clinical study reported that taVNS improved abdominal pain in adolescents with IBS (27). An animal study showed that taVNS ameliorated opioid-induced constipation in rats (28). Unfortunately, these studies did not assess the effects of taVNS on both constipation and abdominal pain. Based on the integrative effects of taVNS on both pain and motility we observed in the animal model of functional dyspepsia and patients with functional dyspepsia (29–31), we hypothesized that taVNS might improve both abdominal pain and constipation in patient with IBS-C by enhancing vagal efferent activity.

The aim of this study was to investigate the effects of taVNS on abdominal pain and constipation, as well as general IBS symptoms in patients with IBS-C and possible autoimmune mechanisms, including inflammatory cytokines and autonomic functions.

## Results

### Patient demographics and baseline characteristics.

**Figure 1:**
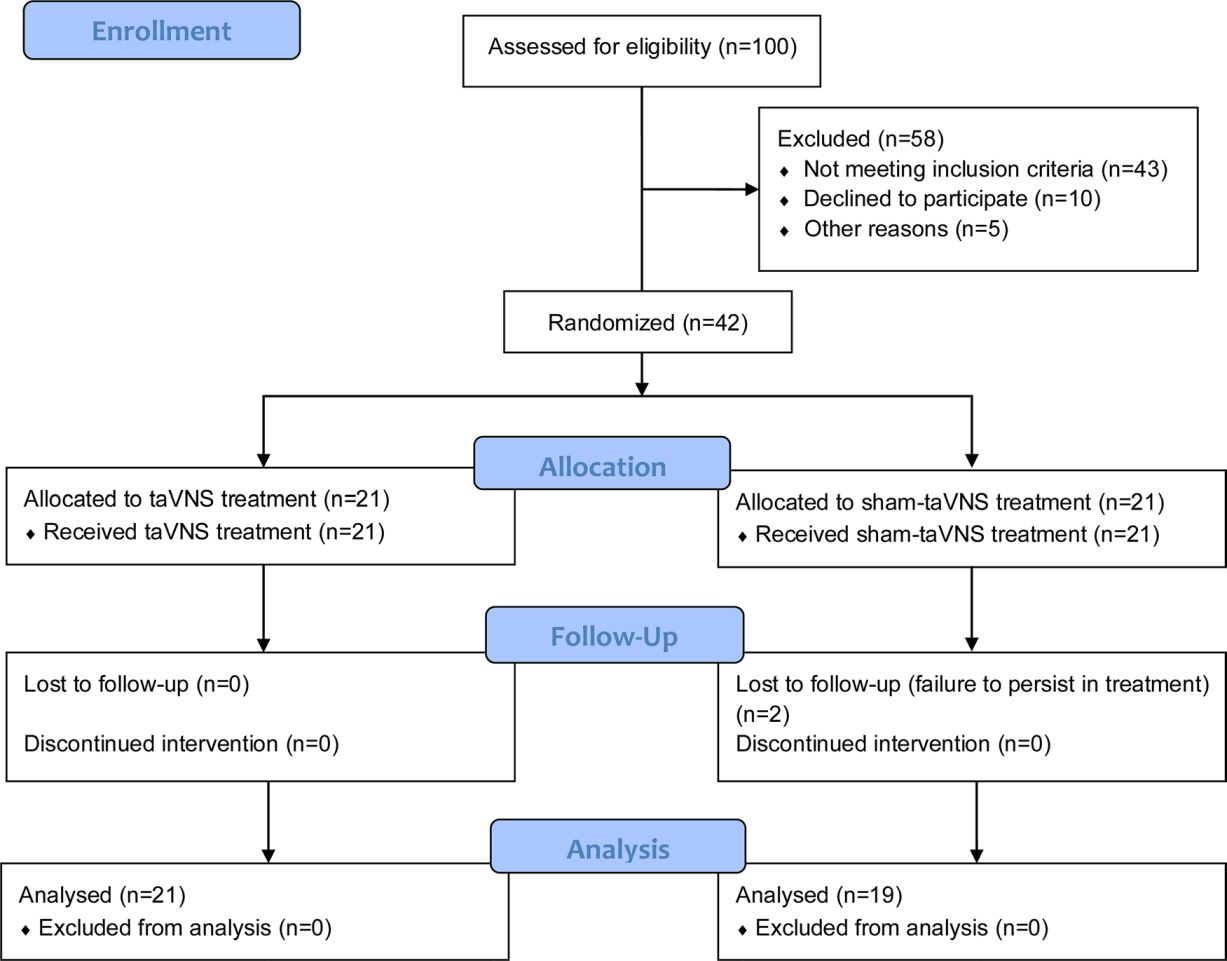
Flowchart of the study design.

In total, 42 IBS-C patients were recruited and randomized into sham-taVNS and taVNS groups with a ratio of 1:1, with 32 female and 10 male patients. Two patients in the sham-taVNS group were dropped in the middle of the study because of failure to persist in treatment (Figure 1). Demographic data and disease characteristics at baseline are shown in Table 1. The demographic data between the 2 groups were matched including sex, age, and BMI. There were no significant differences in disease duration, complete spontaneous bowel movements per week (CSBMs/week), visual analog scale (VAS), IBS quality-of-life questionnaire (IBS-QOL), IBS symptom severity scale (IBS-SSS), Bristol stool form scale (BSFS), self-rating anxiety scale (SAS), and self-rating depression scale (SDS) between the sham-taVNS and taVNS group.

### Effects of taVNS on constipation and pain.

**Figure 2:**
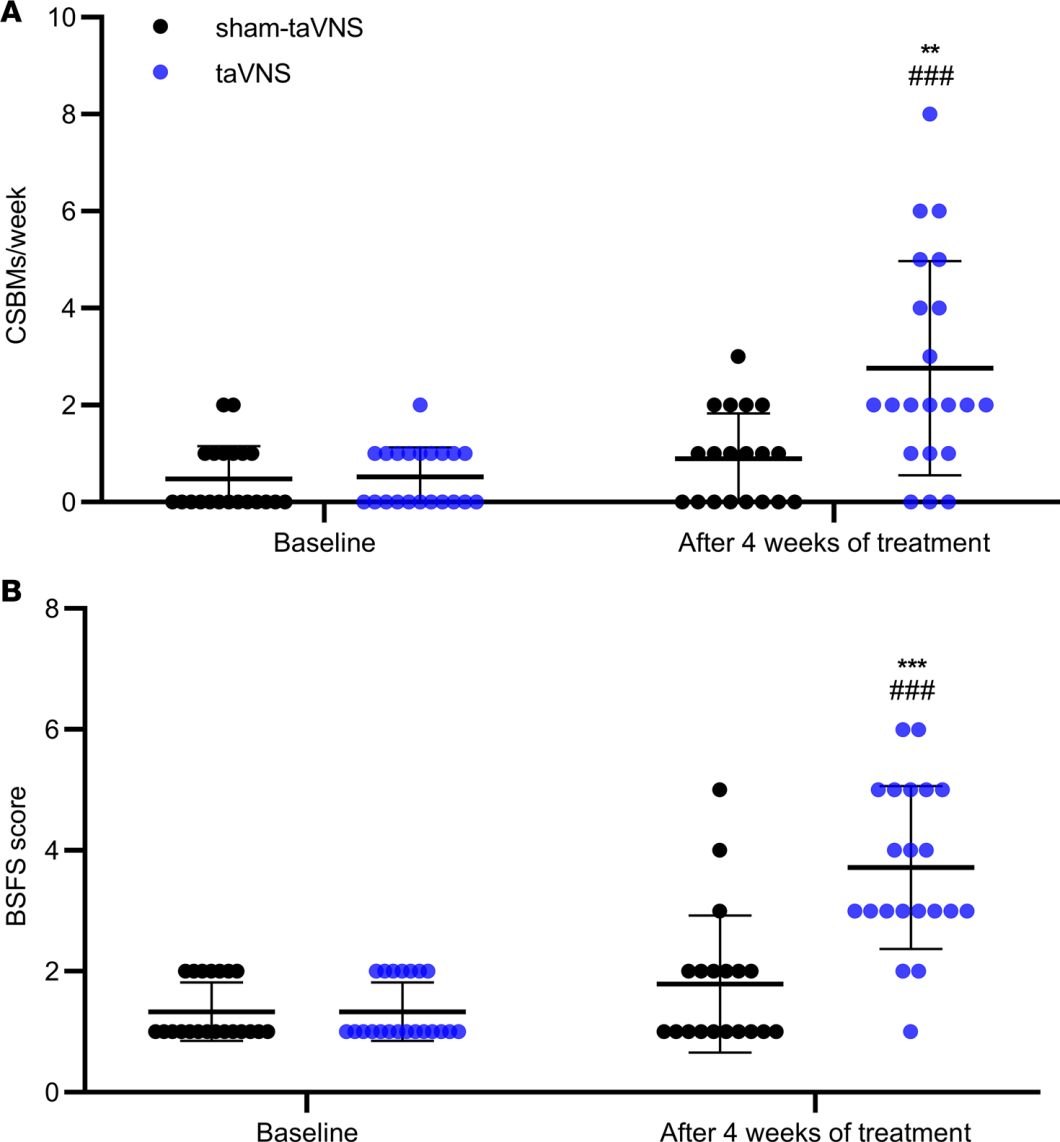
Effects of taVNS on CSBMs/week and BSFS. (**A** and **B**) taVNS increased CSBMs/week (**A**) and BSFS score (**B**). The independent sample *t* test was used to assess the difference between the sham-taVNS and taVNS groups. The paired Student’s *t* test was applied to evaluate the differences before and after the taVNS or sham-taVNS treatment. Mean ± SD is presented (versus sham-taVNS, **P < 0.01, ***P < 0.001; versus baseline, ^###^*P* < 0.001). At baseline, both groups, *n =* 21. After treatment, taVNS group, *n =* 21; sham-taVNS group, *n =* 19.

**Figure 3:**
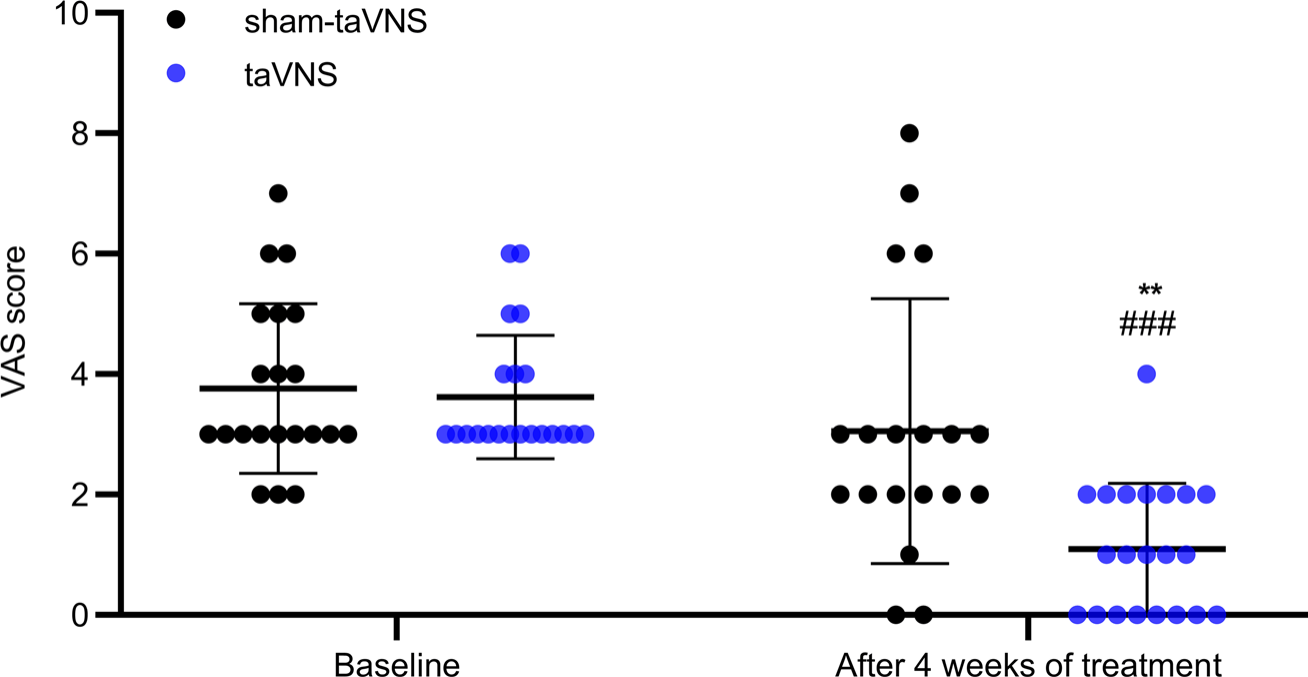
Effects of taVNS on VAS pain score. taVNS decreased VAS score. The independent sample *t* test was used to assess the difference between the sham-taVNS and taVNS groups. The paired Student’s *t* test was applied to evaluate the differences before and after the taVNS or sham-taVNS treatment. Mean ± SD is presented (versus sham-taVNS, **P < 0.01; versus baseline, ^###^*P* < 0.001). At baseline, both groups, *n =* 21. After treatment, taVNS group, *n =* 21; sham-taVNS group, *n =* 19.

As shown in the Figure 2 and Figure 3, the 4-week taVNS treatment substantially improved both constipation and abdominal pain in patients. The number of CSBMs/week was tripled with the taVNS treatment in comparison with the sham-taVNS treatment (0.9 ± 0.9 versus 2.8 ± 2.2, *P =* 0.001). In comparison with the baseline, taVNS increased the number of CSBMs/week by more than 4 fold (0.5 ± 0.6 versus 2.8 ± 2.2, *P* < 0.001; Figure 2A). At the end of taVNS, 18 patients (85.7%) reported to have an increase of ≥ 1 CSBM/week, while only 7 patients (36.8%) reported this after the sham-taVNS treatment (χ^2^ = 10.165, *P* = 0.001). In addition to improving constipation, taVNS also reduced the total dosage of emergency laxatives usage in comparison with sham-taVNS from the first treatment week to the end (Table 2).

The improvement in constipation was also supported by the BSFS. The BSFS score in the taVNS group was significantly higher than that in the sham-taVNS group (1.8 ± 1.1 versus 3.7±1.3, *P* < 0.001); taVNS but not sham-taVNS increased BSFS score in comparison with baseline (1.3 ± 0.5 versus 3.7±1.3, *P* < 0.001; Figure 2B). Before the treatment, all patients showed abnormally hard stools (type 1 and type 2 stools). After the 4-week treatment, the abnormal hard stools were reduced to 14% in the taVNS group and 84% in the sham-taVNS group (χ^2^ =16.243, *P* < 0.001).

Concurrently and importantly, the 4-week taVNS reduced the VAS pain score by 64% compared with the sham-taVNS (3.1 ± 2.2 versus 1.1 ± 1.1, *P* = 0.001) and by 69% compared with the baseline (3.6 ± 1.0 versus 1.1 ± 1.1, *P* < 0.001; Figure 3). At the end of taVNS, 20 patients (95.2%) reported to have an improvement from baseline of ≥ 30% in the weekly average of daily scores, while only 7 patients (36.8%) reported so after the sham-taVNS treatment (χ^2^ = 15.506, *P* < 0.001). Moreover, taVNS also reduced the total dosage of emergency antispasmodic medicine usage in comparison with sham-taVNS from the first treatment week to the end (Table 3). In addition, after the 4 weeks of treatment, the responder rate was 81.0% (17 of 21) in the taVNS group but 26.3% (5 of 19) in the sham-taVNS group (χ^2^ =12.031, *P* = 0.001).

### Effects of taVNS on IBS symptoms and quality of life.

**Figure 4:**
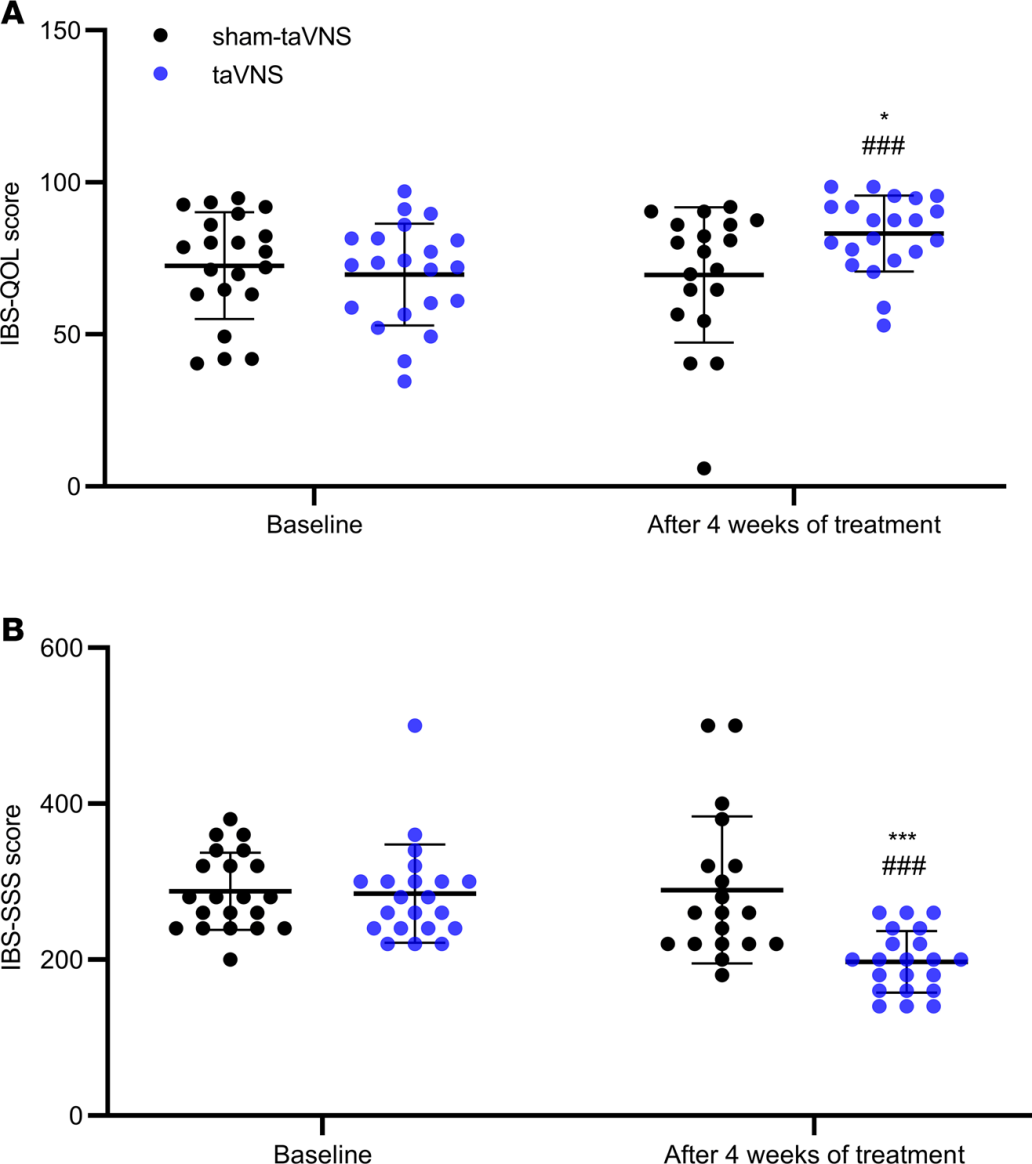
Effects of taVNS on IBS-QOL scores and IBS-SSS. (**A** and **B**) taVAS increased IBS-QOL score (**A**) and decreased IBS-SSS (**B**). The independent sample *t* test was used to assess the difference between the sham-taVNS and taVNS groups. The paired Student’s *t* test was applied to evaluate the differences before and after the taVNS or sham-taVNS treatment. Mean ± SD is presented (versus sham-taVNS, *P < 0.05, ***P < 0.001; versus baseline, ^###^*P* < 0.001). At baseline, both groups, *n =* 21. After treatment, taVNS group, *n =* 21; sham-taVNS group, *n =* 19.

There were no significant differences in IBS-QOL scores and IBS-SSS between the sham-taVNS group and the taVNS group before the treatment (Figure 4). After 4 weeks of treatment, the IBS-QOL score in the taVNS group was significantly higher than that in the sham-taVNS group (69.5 ± 21.2 versus 83.2 ± 12.5, *P* = 0.020); taVNS — not sham-taVNS — increased IBS-QOL score in comparison with baseline (69.7 ± 16.8 versus 83.2 ± 12.5, *P* < 0.001; Figure 4A). The IBS-SSS in the taVNS group was significantly lower than that in the sham-taVNS group (289.5 ± 94.4 versus 197.1 ± 39.6, *P* = 0.001). Moreover, taVNS — not sham-taVNS — decreased IBS-SSS in comparison with baseline (284.8 ± 63.2 versus 197.1 ± 39.6, *P* < 0.001; Figure 4B).

**Figure 5:**
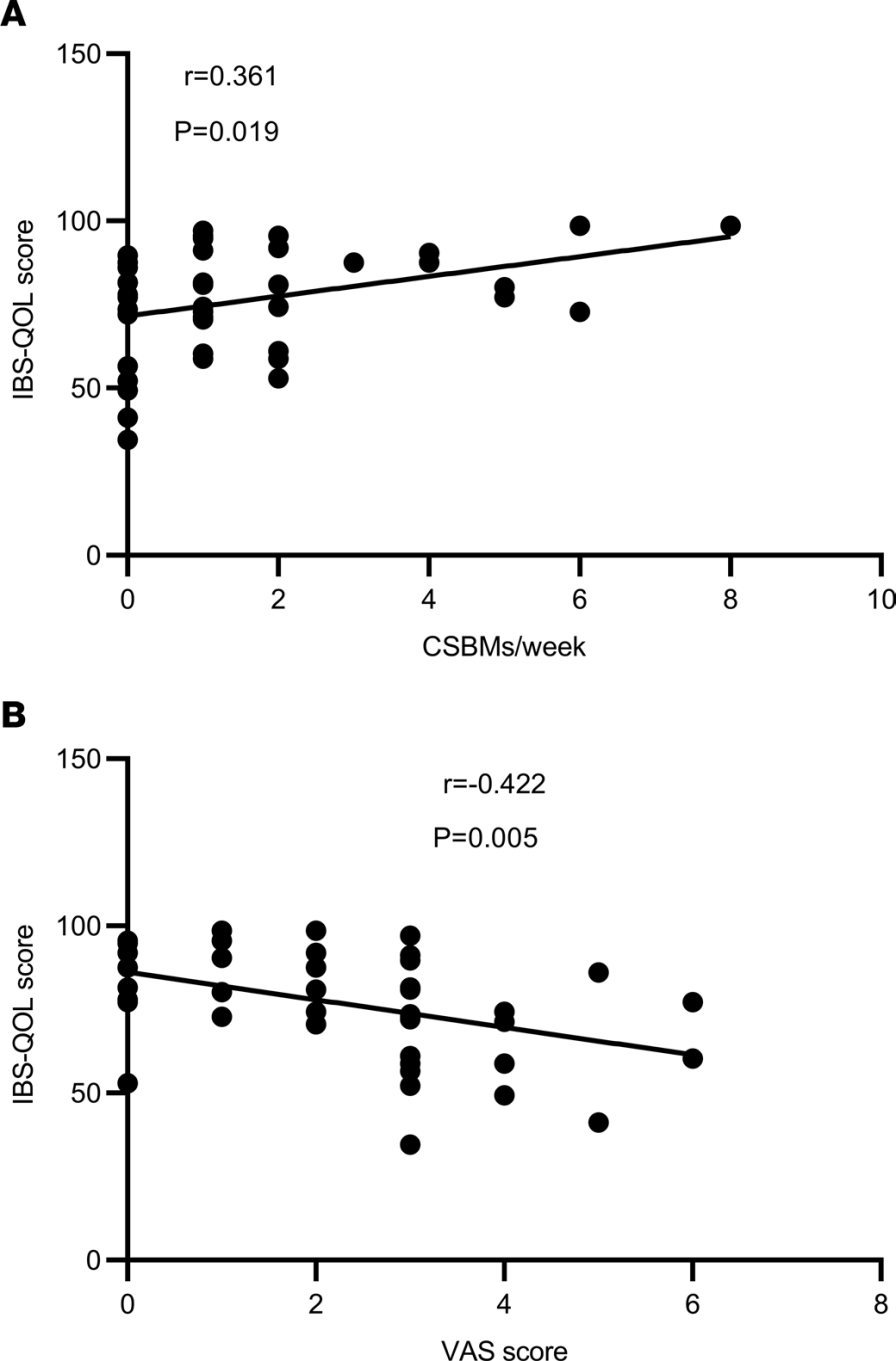
The correlation of IBS-QOL score with CSBMs/week and VAS score in the taVNS group. (**A** and **B**) IBS-QOL score was positively correlated with CSBMs/week (**A**) and negatively correlated with VAS score (**B**). Pearson’s correlation analysis was performed to determine the correlation of IBS-QOL, CSBMs/week, and VAS pain score (before and after treatment, *n =* 42).

Interestingly, the IBS-QOL score was positively correlated with CSBMs/week (*r* = 0.361; *P* = 0.019) and negatively correlated with the VAS pain score (*r* = –0.422; *P* = 0.005; Figure 5).

### Effects of taVNS on anxiety and depression.

**Figure 6:**
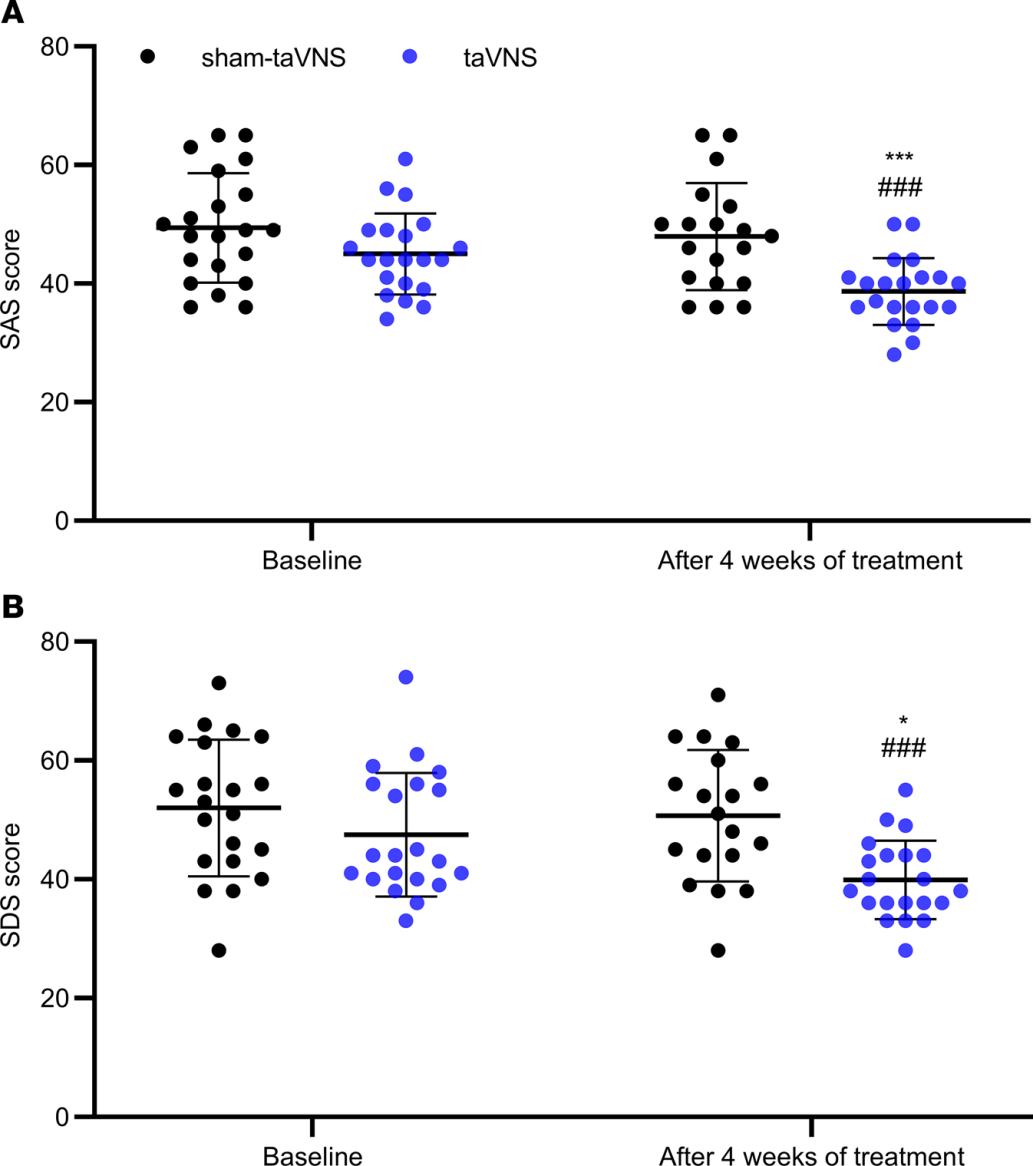
Effects of taVNS on SAS and SDS scores. (**A** and **B**) taVAS decreased SAS (**A**) and SDS score (**B**). The independent sample *t* test was used to assess the difference between the sham-taVNS and taVNS groups. The paired Student’s *t* test was applied to evaluate the differences before and after the taVNS or sham-taVNS treatment. Mean ± SD is presented (versus sham-taVNS, *P < 0.05, ***P < 0.001; versus baseline, ^###^*P* < 0.001). At baseline, both groups, *n =* 21. After treatment, taVNS group, *n =* 21; sham-taVNS group, *n =* 19.

As shown in the Figure 6, after the treatment, taVNS improved anxiety and depression. The SAS and SDS scores in the taVNS group were significantly lower than those in the sham-taVNS group (47.9 ± 9.0 versus 38.7 ± 5.6, *P* < 0.001, and 50.7 ± 11.1 versus 42.6 ± 8.1, *P* = 0.011, respectively), and those at baseline (45.0 ± 6.9 versus 38.7 ± 5.6, *P* < 0.001, and 47.5 ± 10.4 versus 42.6 ± 8.1, *P* < 0.001, respectively; Figure 6, A and B).

### Effects of taVNS on anorectal function.

After 4 weeks of treatment, taVNS significantly improved rectoanal inhibitory reflex (RAIR) and rectal sensation in patients with IBS-C (Table 4). taVNS decreased the volume of distention required to elicit RAIR from 30.0 ± 10.5 mL at baseline to 21.4 ± 4.8 mL (*P* = 0.001), which was significantly different from sham-taVNS treatment (27.4 ± 8.7 mL versus 21.4±4.8 mL, *P* = 0.014). Similarly, there were substantial reductions compared with baseline in first sensation (49.5 ± 31.7 mL versus 34.3 ± 12.1 mL, *P* = 0.020), desire of defecation (121.0 ± 45.4 mL versus 104.8 ± 28.7 mL, *P* = 0.033), and maximum tolerable volume (152.9 ± 49.6 mL versus 131.0 ± 32.8 mL, *P* = 0.029).

Moreover, the threshold volume was significantly lower with taVNS treatment than with sham-taVNS treatment for the first sensation (*P* = 0.005), desire of defecation (*P* = 0.019), and maximum tolerance (*P* = 0.033). Neither taVNS nor sham-taVNS had an effect on the percentage of relaxation during push. In addition, the VAS score was weakly correlated with the first-sense volume (*r* = 0.320; *P* = 0.039) and the maximal tolerable volume (*r* = 0.321; *P* = 0.038), while CSBMs/week showed no correlation with them.

### Effects of taVNS on inflammatory cytokines, serotonin (5-hydroxytryptamine [5-HT]), and calcitonin gene related peptide (CGRP).

**Figure 7:**
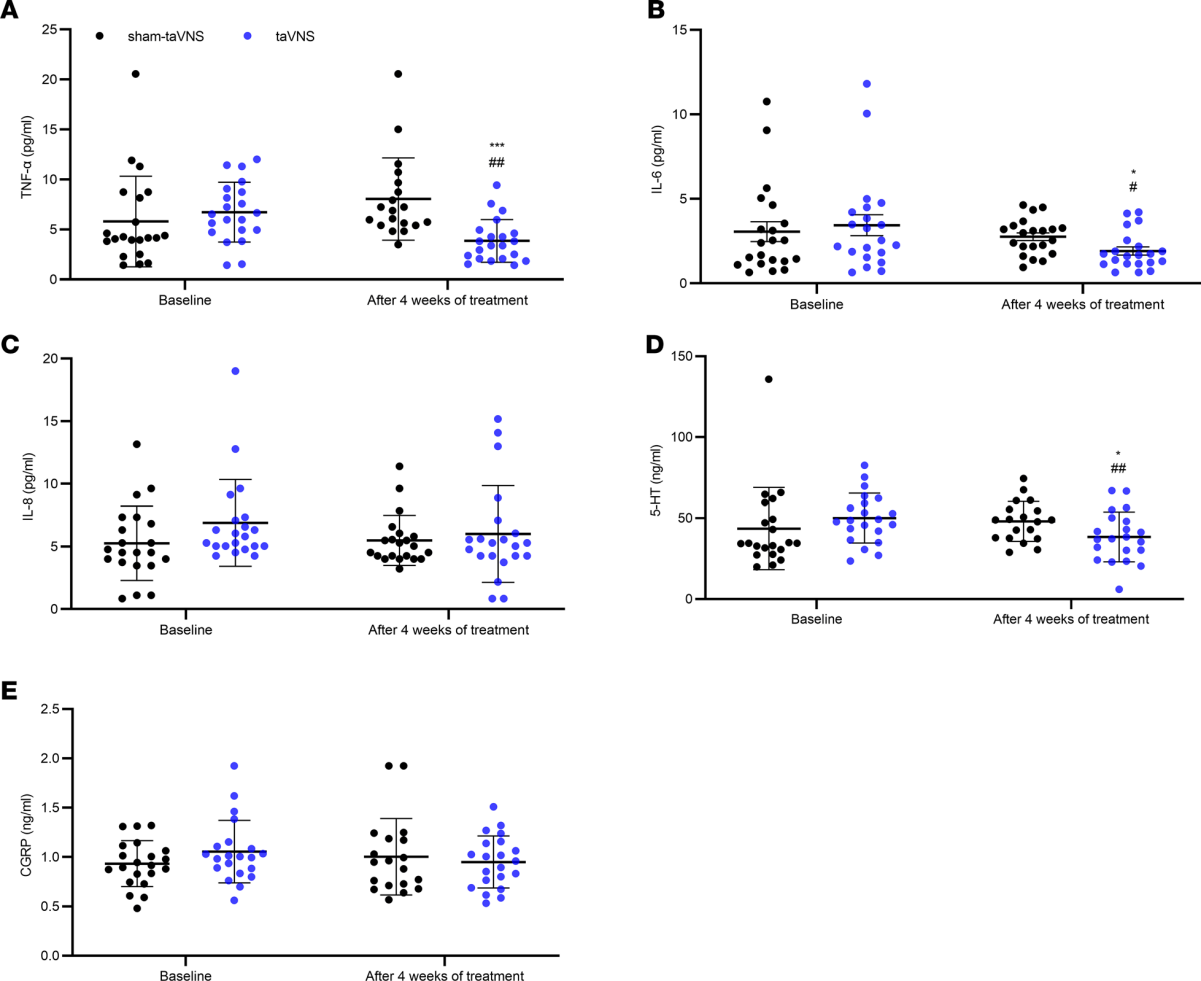
Effects of taVNS on inflammatory cytokines, 5-HT, and CGRP. (**A**, **B**, and **D**) taVNS decreased serum TNF-α (**A**), IL-6 (**B**), and plasma 5-HT (**D**). (**C** and **E**) No significant changes were found in serum IL-8 (**C**) and plasma CGRP (**E**). The independent sample *t* test was used to assess the difference between the sham-taVNS and taVNS groups. The paired Student’s *t* test was applied to evaluate the differences before and after the taVNS or sham-taVNS treatment. Mean ± SD is presented (versus sham-taVNS, *P < 0.05, ***P < 0.001; versus baseline, ^#^*P* < 0.05, ^##^*P* < 0.01). At baseline, both groups, *n =* 21. After treatment, taVNS group, *n =* 21; sham-taVNS group, *n =* 19.

taVNS but not sham-taVNS decreased the serum level of TNF-α, IL-6, and plasma 5-HT (Figure 7). The 4-week taVNS treatment decreased the serum level of TNF-α from 6.7 ± 3.0 pg/mL at baseline to 3.9 ± 2.1 pg/mL (*P* = 0.001) and the serum level of IL-6 from 3.4 ± 2.8 pg/mL at baseline to 1.9 ± 1.1 pg/mL (*P* = 0.037); these post-taVNS values were also significantly lower than those after the sham-taVNS (8.0 ± 4.1 pg/mL versus 3.9 ± 2.1 pg/mL, *P* < 0.001, and 2.7 ± 1.1 pg/mL versus 1.9 ± 1.1 pg/mL, *P* = 0.019, respectively; Figure 7, A and B). Concurrently, after 4 weeks of the treatment, taVNS decreased plasma level of the plasma 5-HT level compared with the baseline (50.0 ± 15.4 ng/mL versus 38.5 ± 15.4 ng/mL, *P* = 0.007), as well as compared with the sham-taVNS treatment (48.0 ± 12.3 ng/mL versus 38.5 ± 15.4 ng/mL, *P* = 0.038; Figure 7D).

No significant difference was noted in the serum level of IL-8 and plasma CGRP level between the taVNS group and sham-taVNS group (Figure 7, C and E).

**Figure 8:**
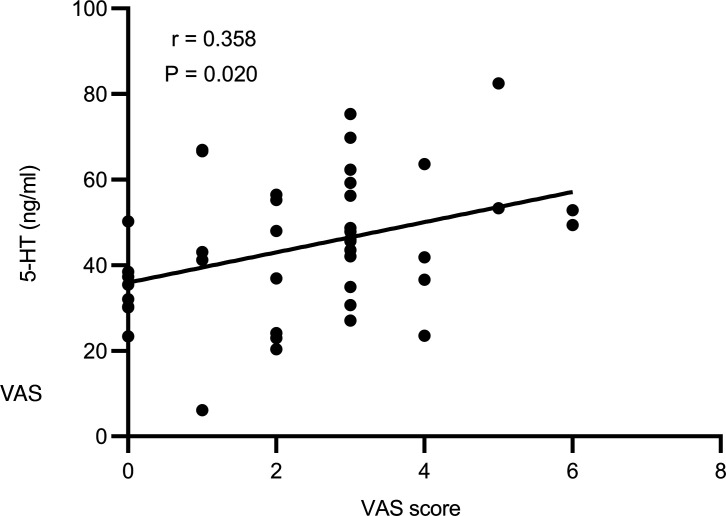
The correlation between VAS score and plasma 5-HT level in the taVNS group. VAS score was positively correlated with plasma 5-HT level. Pearson’s correlation analysis was performed to determine the correlation between VAS pain score and plasma 5-HT level (before and after treatment, *n =* 42).

Interestingly, the plasma level of 5-HT was positively correlated with the VAS pain score (*r* = 0.358; *P* = 0.020; Figure 8) but had no correlation with the first-sense volume and the maximal tolerable volume.

### Mechanisms of taVNS involving autonomic functions.

**Figure 9:**
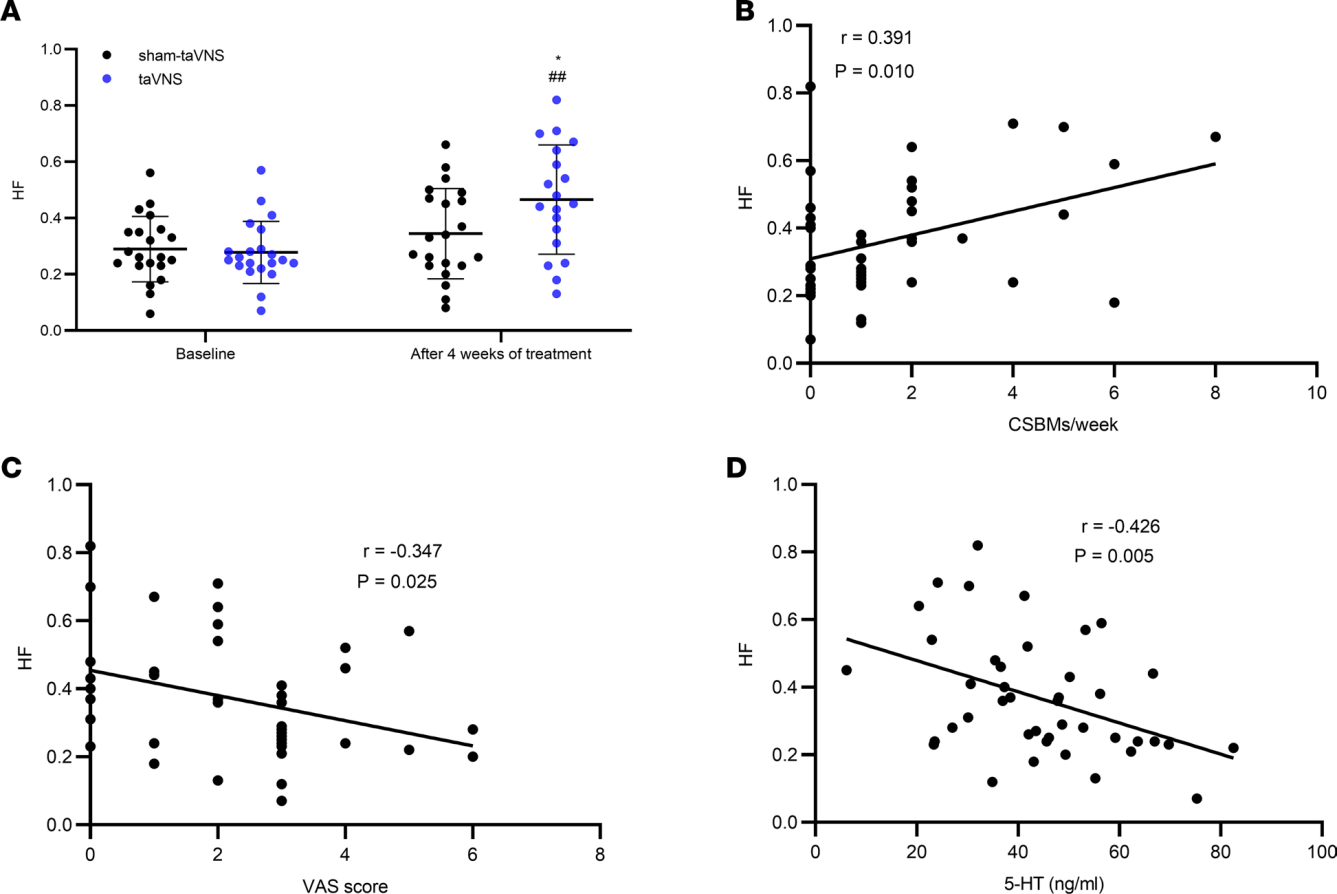
Effects of taVNS on autonomic function and its correlation with CSBMs/week, VAS score, and plasma 5-HT level. taVNS enhanced vagal activity compared with sham-taVNS, as well as compared with baseline after treatment (**A**). (**B**–**D**) HF was positively correlated with CSBMs/week (**B**) and negatively correlated with VAS score (**C**) and plasma 5-HT level (**D**). The independent sample *t* test was used to assess the difference between the sham-taVNS and taVNS groups. The paired Student’s *t* test was applied to evaluate the differences before and after the taVNS or sham-taVNS treatment. Mean ± SD is presented (versus sham-taVNS, *P < 0.05; versus baseline, ^##^*P* < 0.01). At baseline, both groups, *n =* 21. After treatment, taVNS group, *n =* 21; sham-taVNS group, *n =* 19. Pearson’s correlation analysis was performed to determine the correlation of the autonomic functions and CSBMs/week, VAS, and 5-HT in the taVNS group (before and after treatment, *n =* 42). HF, high-frequency band.

taVNS, but not sham-taVNS, increased the vagal activity (high frequency [HF]; Figure 9). At the end of the 4 weeks of the treatment, the vagal activity in the taVNS group was significantly higher than in the sham-taVNS group (0.34 ± 0.17 versus 0.46 ± 0.19, *P* = 0.040) and that at baseline (0.28 ± 0.11 versus 0.46 ± 0.19, *P* = 0.001; Figure 9A).

Interestingly, the HF after the taVNS treatment was weakly correlated with the CSBMs/week (*r* = 0.391; *P* = 0.010; Figure 9B), the VAS pain score (*r* = –0.347; *P* = 0.025; Figure 9C), and the plasma 5-HT level (*r* = –0.426; *P* = 0.005; Figure 9D), suggesting mechanistic roles of the vagal efferent activity.

## Discussion

In this study, we found that the 4-week taVNS treatment substantially ameliorated main symptoms of constipation (increased the number of CSBMs/week and stool form, and decreased the dosage of laxative) and abdominal pain (decreased VAS score and the use of antispasmodic drug), resulting in improvement in overall IBS symptoms and quality of life. Meanwhile, the taVNS treatment also improved symptoms of anxiety and depression. We also found that, physiologically, the taVNS improved rectal sensation associated with defection and rectal anal inhibitory reflex, and that mechanistically, taVNS decreased the serum levels of TNF-α and IL-6 and plasma level of 5-HT; it also enhanced vagal activity assessed by the spectral analysis of heart rate variability (HRV).

Substantial improvement was noted in major symptoms of constipation and abdominal pain with the noninvasive treatment of taVNS in the present study. The taVNS increased the number of CSBMs/week by more than 4 fold in comparison with the baseline and 2 fold in comparison with the sham-taVNS treatment; it also reduced the percentage of abnormally hard stools by 86% in comparison with the baseline and 81% in comparison with the sham-taVNS, and it decreased the use of laxatives. These findings demonstrated a substantial improvement in constipation. Meanwhile and importantly, the taVNS reduced the VAS pain score by 64% compared with the sham-taVNS and by 69% compared with the baseline, and it decreased the usage of antispasmodic drugs, suggesting an effective analgesic effect. Additionally, we also found that the responder rate with the taVNS was 81.0%, which was higher than that with some pharmacotherapies, such as linaclotide (56.3%; ref. 32). Previously, Zhang et al. (28) showed that both VNS and taVNS enhanced colon motility and improved opioid-induced constipation in rats. Li et al. (33) reported that auricular acupuncture (the stimulation site located in auricular branch of vagus nerve) increased gastrointestinal transit in rats. In human studies, A review (34) reported that auriculotherapy might be used as a complementary therapy for constipation by stimulating the external surface of the auricle. Additionally, VNS and taVNS have been shown effective in relieving pain in both animals and humans (24, 35–37). In animal studies, Chen et al. (38) demonstrated that VNS reduced visceral pain to standardized mechanical visceral distension in vagotomized rats. Guo et al. (36) found that taVNS reduced pain intensity in rats with depression-chronic somatic pain comorbidity. In clinical studies, Multon and Schoenen showed a clear antinociceptive effect of VNS in acute or inflammatory pain with different stimulation protocols (24). A large randomized clinical trial showed that taVNS ameliorated abdominal pain in patients with functional abdominal pain disorders and had sustained efficacy in adolescents (27). The potential novelty of our findings was that taVNS was not only able to improve constipation, but it was also able to reduce abdominal pain in patients with IBS-C.

In addition, after the 4 weeks of taVNS treatment, the overall IBS symptoms were also improved, including abdominal pain intensity and frequency, abdominal distension, defecation satisfaction, and the quality of life with a decrease in the IBS-SSS and an increase in IBS-QOL score, probably attributed to the improvement in constipation and abdominal pain, since our data show that the IBS-QOL score was positively, although weakly, correlated with the CSBMs/week and negatively correlated with the VAS pain score. Some scholars have reported a correlation between gastrointestinal symptoms and mental states in IBS patients with depression and anxiety (39–41). In the present study, the taVNS improved anxiety and depression, reflected as a decrease in the SAS and SDS scores, consistent with previous studies (42, 43); this was probably attributed to the improvement of IBS symptoms.

Dyssynergic defecation, including motor abnormalities and sensory dysfunction, is common and affects up to one-half of patients with constipation (44). A previous study reported the impairment of RAIR, and the volume of distention eliciting a relaxation of 50% was higher in patients with constipation compared with healthy subjects (45). Studies have also shown that the rectal distention threshold for both the first sensation and the desire to defecate was higher in about 60% of patients with dyssynergic defecation (46, 47). In the current study, we found an enhancive effect of taVNS on rectal sensation in patients with IBS-C, consistent with previous studies with the use of electrical acupuncture or transcutaneous electrical acupuncture (48, 49). Kenefick et al. reported that electrical acupuncture decreased the rectal distention threshold for the urge to defecate and maximum tolerance in patients with idiopathic constipation (48). Zhang et al. demonstrated that transcutaneous electrical acustimulation significantly decreased the volume of rectal distention required to elicit RAIR, and it also decreased the threshold volume of rectal distention for the first sensation and the maximum tolerance in patients with constipation (49). In the present study, we found that the taVNS decreased both the rectal distention volume to elicit RAIR and the threshold volume of rectal distention for the first sensation, desire of defecation, and maximum tolerance. The taVNS-induced improvement in rectal sensation might have contributed to the improvement in symptoms of constipation.

Inflammation and cytokine imbalance may act as potential etiological factors in IBS (50, 51). In the current study, the taVNS decreased serum TNF-α and IL-6 after 4 weeks of treatment. In a previous study, needle-based VNS decreased the plasma level of TNF-α and IL-6 in a rodent model of 2,4,6-trinitrobenzene sulfonic acid–induced (TNBS-induced) colitis by inhibiting proinflammatory cytokines via the autonomic mechanism (52). In clinical studies, needle-based auricular VNS decreased serum IL-6 in patients with lung lobectomy (53); taVNS decreased serum TNF-α in patients with paroxysmal atrial fibrillation (54). Matteoli et al. (23) suggested that VNS reduced intestinal inflammation through α7 nicotinic acetylcholine receptor (α7nAChR) activity in the intestine muscularis–resident macrophages in a mouse model of postoperative ileus. Wang et al. found that electrical stimulation of the vagus nerve inhibited TNF-α synthesis in WT endotoxemia mice via α7nAChR subunit activity (55). Zhao et al. (56) showed that taVNS strongly inhibited LPS-induced proinflammatory cytokines, including TNF-α and IL-6 via the α7nAChR-mediated cholinergic antiinflammatory pathway in endotoxemia rats. Accordingly, we speculated that taVNS decreased TNF-α and IL-6 by activating the cholinergic antiinflammatory pathway.

Dysfunction of the brain-gut axis is also an important cause of IBS. The brain-gut axis is a complex network linking the gastrointestinal tract to the CNS, which includes the central and the autonomic nervous systems, the enteric nervous system, and the neuroendocrine and neuroimmune systems (57). Neuroendocrine transmitters serve as bridges and modulatory functions in the brain gut axis. In our study, the taVNS decreased plasma 5-HT after 4 weeks of treatment in comparison with baseline, and interestingly, the plasma level of 5-HT was positively correlated with the VAS score. 5-HT is a classic pain-related substance (58). Clinical studies have shown an elevated level of 5-HT in the mucosa of the gastrointestinal tract in IBS-C patients (59) and increased 5-HT release, contributing to the development of abdominal pain in IBS (60). An animal study showed that the analgesic effect of quercetin on postinflammatory IBS might result from a reduction of 5-HT availability in the colon (61). Accordingly, we speculated that taVNS alleviated visceral pain through the 5-HT pathway.

Surprisingly, the current study failed to show an increase of serum IL-8 and plasma CGRP. This could be attributed to the fact that the levels of IL-8 and CGRP measured in this study were comparable with those in healthy controls (62, 63).

The effects of the taVNS therapy on IBS-C observed in this study might be secondary to changes in autonomic functions. It is well known that gastrointestinal motility is enhanced by vagal activation and/or sympathetic suppression, and that the autonomic neurological dysfunction plays an important role in the progression of impaired gastrointestinal motility (64). Previous studies have reported that VNS and taVNS enhanced the activation of the parasympathetic nervous system and decreased the activation of the sympathetic nervous system (65, 66). In animals, VNS and taVNS have been reported to improve gastrointestinal dysmotility by enhancing vagal activity and decreasing sympathetic activity (28, 30). In a clinical study, taVNS was noted to enhance gastroduodenal motility by increasing vagal tone (67).

Visceral hypersensitivity is an important cause of chronic abdominal pain in functional gastrointestinal diseases, and the vagus nerve exerts an antinociceptive effect within the viscera (68). VNS was reported to decreased nociceptive behaviors in a rodent model of visceral hypersensitivity by increasing vagal afferent excitability (69). Auricular electroacupuncture improved gastric hypersensitivity in animals by increasing vagal activity and decreasing sympathovagal activity (31). Similarly, taVNS prevented the development of acid-induced oesophageal hypersensitivity and increased pain-tolerance thresholds in patients by enhancing vagal tone (70, 71). In the present study, using the spectral analysis of HRV (72), we found that taVNS increased vagal activity (HF). The vagal nerve activity was positively but weakly correlated with the CSBMs/week, and it was negatively correlated with the VAS score. These interesting findings suggested, again, the autonomic mechanisms involved in the improvement of IBS-C symptoms with the taVNS. We further speculated that taVNS enhanced vagal activity and resulted in improvement in colon motility and visceral hypersensitivity, leading to a reduction in main constipation symptoms and abdominal pain. While the exact neural pathway was not investigated in the current study, previous animal studies (73, 74) have reported that manual or electrical stimulation at the auricular acupoints innervated by the auricular branch vagus nerve activated neurons in the nucleus of the solitary tract (NTS) via the projection from the auricular vagal nerve to the NTS, which led to a central response in the dorsal motor nucleus of the vagus, resulting in enhanced vagal efferent activity to the gastrointestinal tract (75).

The improvement in rectal sensation with the taVNS was puzzling, as the vagus nerve does not innervate the rectum, and possible mechanisms deserve further investigation. Similar results were also observed in a separate study in which auricular VNS was found to improve opioid-induced constipation in and accelerate whole colon transit (76). We speculated that this might be attributed to (a) possible innervation of the vagus nerve to the rectum and/or (b) a possible vagal afferent and sacral efferent pathway. In a few recent studies, sacral nerve stimulation was reported to alter functions of the gastrointestinal organs (stomach and small intestine) that are not innervated with the sacral nerve (77, 78), and a spinal afferent and vagal efferent pathway was indicated with the sacral nerve stimulation (79).

There were several limitations in this study: (a) this was a single-center and small sample size study; (b) the patients were not classified into normal or slow colon transit; and (c) there was no long-term follow-up observation. However, a previous multicenter, randomized, parallel, sham-controlled trial reported that the ameliorating effect of electroacupuncture on constipation was sustained for at least 12 weeks after the termination of the therapy in patients with chronic severe functional constipation (80). Accordingly, we would speculate a similar sustained effect with the taVNS treatment in patients with IBS-C; however, further studies are needed.

In conclusion, noninvasive taVNS improves both constipation and abdominal pain in patients with IBS-C. The improvement in IBS-C symptoms might be attributed to the integrative effects of taVNS on intestinal functions mediated via the autoimmune mechanisms.

## Methods

### Study participants

In this single-center, single-blind, randomized controlled trial (RCT), patients were allocated (1:1) to undergo either taVNS or sham-taVNS treatment. Forty-two patients with IBS-C were recruited into this study.

Inclusion criteria were as follows: (a) aged 18–75 years, (b) willing to sign a written informed consent form, and (c) met the Rome IV diagnostic criteria (1) for IBS-C. Exclusion criteria included: (a) a history of previous abdominal surgery (other than appendectomy); (b) the presence of carcinoma; (c) any organic diseases causing constipation or neurologic diseases such as multiple sclerosis, rachischisis, Parkinson’s disease, or spinal cord injury; (d) taking antidepressant agents including tricyclic antidepressants and selective serotonin reuptake inhibitors; (e) a serious concomitant disease of the heart, liver, kidney, or diabetes; (f) pregnancy or lactation; (g) participating in another trial or enrolled in a trial during the past month; or (h) an allergic reaction to surface electrodes.

### Study design and protocol

Enrolled patients were randomly divided into 2 groups (taVNS and sham-taVNS) with a ratio of 1:1 according to a computer-generated random digital table. The sample size was calculated by G*power analyses based on our preliminary study (49), in which the main outcome was CSBMs/week. The average value of CSBMs in the control group was 2.3 times/week with a SD ± 2.1, and the average value of CSBMs in the treatment group was 3.7 times/week with a SD ± 1.4. The calculated effect size d equaled 0.784. With an α level of 5%, a sample size of 42 (2 × 21) patients was required to ensure a statistical power of 80% in the 1-tailed *t* test (difference between 2 dependent means [2 groups]). In addition, the sample size was also calculated by G*power analyses based on another study (81), in which VAS pain score was used as an outcome measurement. The average VAS pain score in the control group was 2.71 with a SD ± 2.57; the average pain score in the treatment group was 0.95 with a SD ± 0.84. The calculated effect size d equaled 0.921. With an α level of 5% (1-tailed), a sample size of 32 (2 × 16 samples) patients was required to ensure a statistical power of 80% in the *t* test (difference between 2 dependent means [2 groups]). Then, we took the larger one (42 samples) as the sample size of this study.

One week before the start of the study, patients were asked to stop all constipation and IBS medications, including laxatives, prokinetic agents, probiotics, and antispasmodics. After a 1-week run-in period, patients in each group were requested to complete BSFS, VAS pain score, IBS-SSS, IBS-QOL, SAS, and SDS followed by HRV and high-resolution anorectal manometry (HRAM) tests at baseline. The chronic taVNS and sham-taVNS intervention was performed twice a day (8 a.m. and 8 p.m.) for 30 minutes each time, lasting for 4 weeks.

During the run-in and treatment periods, only macrogol 4000 powder (Forlax) and pivinium bromide tablets (Dicetel) were permitted for use when the patient could not tolerate symptoms of constipation and abdominal pain, with the use of the medications and their dosage recorded. The patients were requested to complete BSFS, VAS, IBS-SSS, IBS-QOL, SAS, and SDS every week and fill out the bowel diary during taVNS or sham-taVNS to record the frequency of defecation, time of defecation, stool quality, difficulty degree of defecation, and feeling of complete emptying of stool. After 4 weeks of treatment, HRV and HRAM were performed again. The study schedule is detailed in Table 5.

### taVNS/sham-taVNS

The taVNS treatment was performed at auricular cymba concha (82–84). One pair of electrodes was placed at bilateral auricular concha, via which trains of pulses were delivered from a watch-size digital stimulator (SNM-FDC01, Ningbo Maida Medical Device Inc.) (Supplemental Figure 1A; supplemental material available online with this article; https://doi.org/10.1172/jci.insight.150052DS1). The stimulation parameters were set as follows: train on-time of 2 seconds and off-time of a 3-second pulse width of 0.5 ms, pulse frequency of 25 Hz, and amplitude of 0–2 mA (at the maximum level tolerated by the subject). The stimulation parameters we chose were based on a previous study of transcutaneous electrical stimulation via surface electrodes showing both prokinetic and analgesic effects (81).

Sham-taVNS was performed with the same parameters as taVNS except that electrical stimulation was performed at sham points (85, 86). The sham point was at the elbow area (Supplemental Figure 1B). The patients were blinded to the type of treatment.

### Symptom assessment

#### Primary outcome measures.

The complete spontaneous bowel movement was defined as the bowel movement that occurred without use of any medication or other methods to assist defecation in the previous 24 hours and with a feeling of complete evacuation (87). Time of defecation, stool quality, difficulty degree of defecation, and feeling of complete emptying of stool recorded in the bowel diary were documented.

#### VAS pain score.

Each patient rated her abdominal pain from the previous week on a 0–10 scale, with 0 indicating no pain and 10 indicating the worst imaginable pain. According to a previous study (88), the reliability and validity of the VAS in patients with IBS has been established. A patient was considered as a responder if there was an improvement (during the same week for at least 50% of the treatment period weeks) from baseline of ≥ 30% in the weekly average of daily scores for worst abdominal pain and an increase of ≥ 1 CSBM per week from baseline, according to the FDA criteria (32).

### Secondary outcome measures

#### IBS-SSS.

The IBS-SSS questionnaire is a simple method of monitoring IBS and its progress, and it consists of 5 questions (abdominal pain intensity, abdominal pain frequency, abdominal distension degree, defecation satisfaction, and interference with quality of life), summing to a score of 500 points, with a higher score indicating a worse condition. Earlier studies have established that scores < 175 represent mild IBS symptoms, scores 175–300 represent moderate severity, and scores > 300 represent severe IBS (89).

#### IBS-QOL.

The IBS-QOL is composed of 8 dimensions (dysphoria, interference with activity, body image, health concerns, food avoidance, social reaction, sex, and relationships), with 34 items assessing the degree to which IBS interferes with the patient’s quality of life. Each item is evaluated on a 5-point Likert scale (score 5–1). The total score on the IBS-QOL ranges from 34 to 170. The score for each domain can be converted by the formula to give a score on the scale of 0–100, with the converted score = (original score – lowest possible score)/possible score range × 100. Higher scores indicate better quality of life. The scale has achieved high validity and reliability with IBS patients in previous studies (90).

#### BSFS.

The BSFS is a useful tool to evaluate bowel habit and has been widely used in clinical practice and research worldwide. It was recorded as 1–7 points according to stool type 1–7 (from the hardest [type 1] to the softest [type 7]; ref. 1). The lower the score, the more severe the constipation.

#### The Zung self-rating anxiety and depression scale (SAS/SDS).

The SAS and SDS questionnaires were used to assess anxiety and depression symptoms of patients with IBS-C, respectively. Both SAS and SDS are 20-item Likert scales, in which items tap physiological and psychological symptoms and are rated by participants according to how each applied to them within the past week, using a 4-point scale ranging from 1 (none, or a little of the time) to 4 (most, or all of the time) (91). The standard score of 50 was utilized as the critical value to divide depression or anxiety, and higher scores indicate greater severity.

### Assessment of autonomic functions

The autonomic functions were assessed by the spectral analysis of HRV derived from ECG. HRV test was performed during the 2 office visits. After 10 minutes of rest, the patients were requested to lie down, and HRV was recorded for 30 minutes. Five ECG electrodes were placed as follows: 2 grounded electrodes on the junction of the midclavicular line and the bilateral second intercostal space, 2 reference electrodes on the intersection of the midclavicular line and the bilateral eighth rib, and one on the junction of the right sternal border and the third intercostal space (86). All the electrodes were connected to the ECG amplifier (ECG-01A, Ningbo Maida Medical Device Inc.) by 5 differently colored electrical wires. The HRV signal was derived from the ECG via identifying R peaks and determining RR intervals using a special software system developed and validated previously (85, 86). Furthermore, the overall power spectrum of the HRV signal was calculated, as well as the powers of different frequency subbands. The power in the low-frequency band (LF, 0.04–0.15 Hz) reflects mainly sympathetic activity, whereas the power in the high-frequency band (HF, 0.15–0.50 Hz) represents purely parasympathetic or vagal activity. In the present study, the standardized values were used — i.e., LF was calculated as LF/(HF + LF), and HF was calculated as HF/(HF + LF) (85).

### HRAM

Following HRV test, HRAM was undertaken using a solid-state manometric instrument with 12 circumferential sensors spaced at 1 cm intervals and an outer diameter of 4.2 mm (Medical Measurement Systems Inc.) to evaluate anorectal sensorimotor function. Patients were asked to be in the left lateral position with the hips and knees flexed, and then the solid-state catheter was inserted into the rectum through the anal orifice. After a 3-minute period of stabilization, the sequential maneuver procedures are as follows: rest, squeeze, endurance squeeze, push, RAIR, and rectal sensory threshold. Threshold volume of rectal distention for eliciting RAIR was evaluated by rapid inflation of latex balloon in rectum. First-sensation volume, desire to defecate threshold, and maximum tolerance were tested by constant dilation of balloon (92).

### Blood draw and assay

A blood sample was taken at 8 a.m. at baseline and after 4 weeks of treatment. For each patient, about 5 mL of blood was drawn into a procoagulant tube and 4 mL into 2 anticoagulant tubes with EDTA; it was centrifuged at 4°C and 1139*g* for 10 minutes and 5 minutes respectively. The serum in the procoagulant tube was divided into 3 portions, and the plasma in the anticoagulant tubes was divided into 2 portions — each about 0.5 mL — and placed at –80°C for assay within 6 months. Human ELISA kits (Anogen, catalogs EL10008, EL10019, and EL10023) were used for the analysis of IL-6, IL-8, and TNF-α, respectively. Serotonin (5-HT) was analyzed by human ELISA kits (LDN, catalog BA E-8900) and CGRP was analyzed by human ELISA kits (phoenix pep, EK-015-02). All blood assays were performed blindly by BioTNT.

### Statistics

Quantitative variables are reported as mean ± SD, while categorical data qualitative variables are presented as absolute values and percentages. Categorical data were compared using the χ^2^ test. The independent sample 2-tailed *t* test was used to assess the difference between the sham-taVNS and taVNS groups. The paired Student’s *t* test was applied to evaluate the differences before and after the taVNS or sham-taVNS treatment. *P <* 0.05 was considered statistically significant. Pearson’s correlation analysis was used to determine the correlation of the autonomic functions, CSBMs, VAS, and 5-HT. Data were analyzed by statistical software SPSS 23.0.

### Study approval

The study protocol was approved by the Ethics Committee of Shanghai East Hospital Affiliated to Tongji University, and all subjects signed the informed consent form before their participation in the study. Participants are identified by number.

## Author contributions

Study design was contributed by FL, JDZC, and XS. Data acquisition was contributed by WL and YH. Data analysis was contributed by XS and BZ. Manuscript preparation was contributed by XS and FL. Materials and analysis tools were contributed by JDZC and YH.

## Supplementary Material

Supplemental data

Trial reporting checklists

ICMJE disclosure forms

## Figures and Tables

**Table 5:**
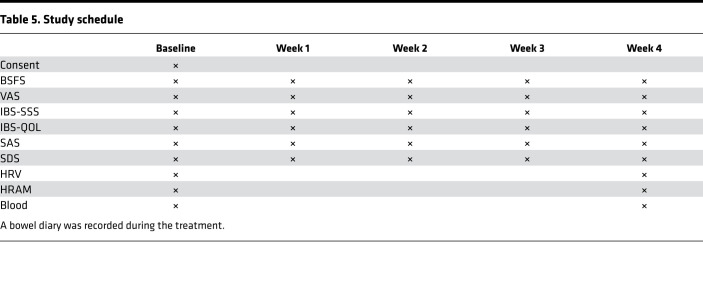
Study schedule

**Table 4:**
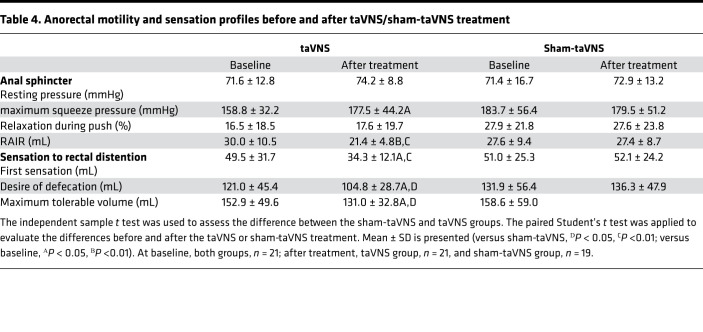
Anorectal motility and sensation profiles before and after taVNS/sham-taVNS treatment

**Table 1:**
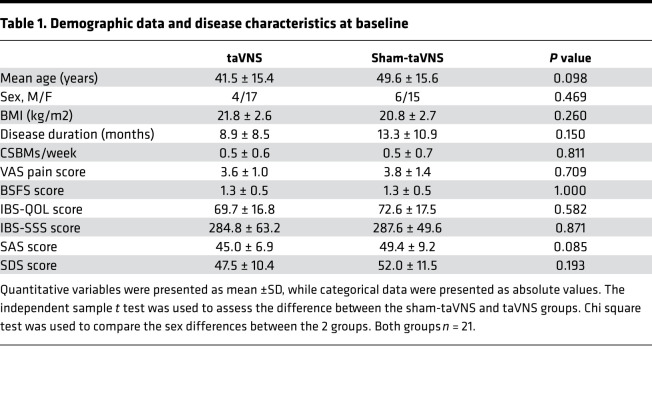
Demographic data and disease characteristics at baseline

**Table 2:**
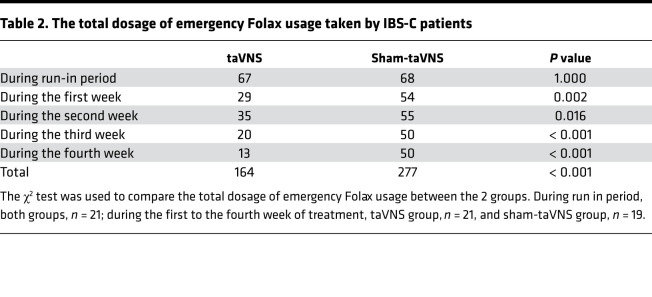
The total dosage of emergency Folax usage taken by IBS-C patients

**Table 3:**
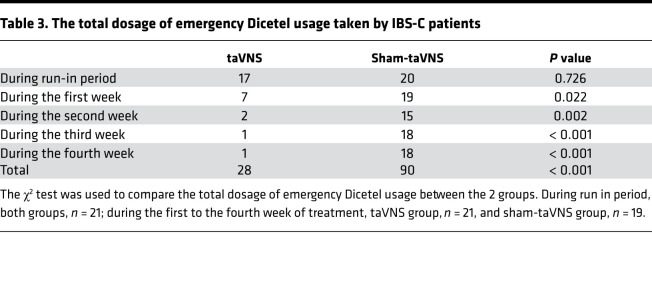
The total dosage of emergency Dicetel usage taken by IBS-C patients

## Version 1 06/17/2021

In-Press Preview

## Version 2 07/22/2021

Electronic publication
