# Supplementary material for: Ameliorating effects and mechanisms of transcutaneous auricular vagal nerve stimulation on abdominal pain and constipation
Source: JCI Insight. 2021 Jul 22;6(14):e150052. doi: 10.1172/jci.insight.150052 (PMC8410029; doi:10.1172/jci.insight.150052)
Supplement: Supplemental data [file jciinsight-6-150052-s101.pdf]

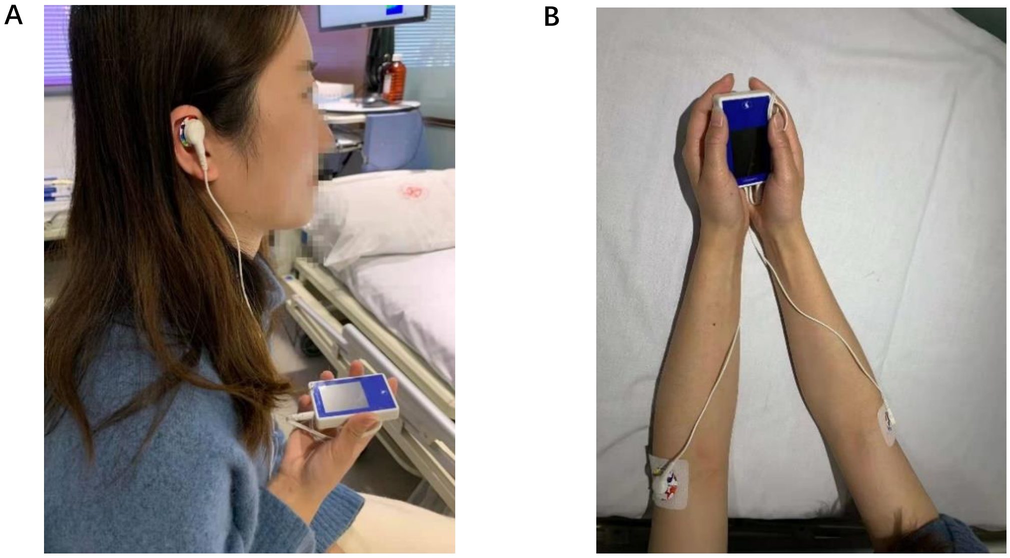

Supplementary Figure 1. taVNS at bilateral cymba concha (A) and sham-taVNS at bilateral elbow area (B). One pair of surface ECG electrodes was applied at stimulation sites. A watch-size digital stimulator was used to deliver electrical stimulation. taVNS, transcutaneous auricular vagal nerve stimulation.
