# Supplementary material for: Ameliorating effects and mechanisms of transcutaneous auricular vagal nerve stimulation on abdominal pain and constipation
Source: JCI Insight. 2021 Jul 22;6(14):e150052. doi: 10.1172/jci.insight.150052 (PMC8410029; doi:10.1172/jci.insight.150052)
Supplement: ICMJE disclosure forms [file jciinsight-6-150052-s103.pdf]

ICMJE DISCLOSURE FORM

Date: ___June-10th-2021____
Your Name: ___ Xiaodan Shi ____
Manuscript Title: ___ Ameliorating effects and mechanisms of transcutaneous auricular vagal nerve stimulation on abdominal pain and constipation ____
Manuscript number (if known): ___ 150052-INS-CMED-RV-3 ____

In the interest of transparency, we ask you to disclose all relationships/activities/interests listed below that are related to the content of your manuscript. "Related" means any relation with for-profit or not-for-profit third parties whose interests may be affected by the content of the manuscript. Disclosure represents a commitment to transparency and does not necessarily indicate a bias. If you are in doubt about whether to list a relationship/activity/interest, it is preferable that you do so.

The following questions apply to the author's relationships/activities/interests as they relate to the current manuscript only.

The author's relationships/activities/interests should be defined broadly. For example, if your manuscript pertains to the epidemiology of hypertension, you should declare all relationships with manufacturers of antihypertensive medication, even if that medication is not mentioned in the manuscript.

In item #1 below, report all support for the work reported in this manuscript without time limit. For all other items, the time frame for disclosure is the past 36 months.

		**Name all entities with whom you have this relationship or indicate none (add rows as needed)**	**Specifications/Comments (e.g., if payments were made to you or to your institution)**
		**Time frame: Since the initial planning of the work**	
1	All support for the present manuscript (e.g., funding, provision of study materials, medical writing, article processing charges, etc.) **No time limit for this item.**	__ √ __None	
		**Time frame: past 36 months**	
2	Grants or contracts from any entity (if not indicated in item #1 above).	__ √ __None	
3	Royalties or licenses	__ √ __None	
4	Consulting fees	__ √ __None	

5	Payment or honoraria for lectures, presentations, speakers bureaus, manuscript writing or educational events	__ √ __None	
6	Payment for expert testimony	__ √ __None	
7	Support for attending meetings and/or travel	__ √ __None	
8	Patents planned, issued or pending	__ √ __None	
9	Participation on a Data Safety Monitoring Board or Advisory Board	__ √ __None	
10	Leadership or fiduciary role in other board, society, committee or advocacy group, paid or unpaid	__ √ __None	
11	Stock or stock options	__ √ __None	
12	Receipt of equipment, materials, drugs, medical writing, gifts or other services	__ √ __None	
13	Other financial or non-financial interests	__ √ __None	

Please place an "X" next to the following statement to indicate your agreement:

__X_ I certify that I have answered every question and have not altered the wording of any of the questions on this form.

ICMJE DISCLOSURE FORM

Date: ___ June-10th-2021____
Your Name: ___ Yedong Hu ____
Manuscript Title: ___ Ameliorating effects and mechanisms of transcutaneous auricular vagal nerve stimulation on abdominal pain and constipation ____
Manuscript number (if known): ___ 150052-INS-CMED-RV-3 ___________________________________

In the interest of transparency, we ask you to disclose all relationships/activities/interests listed below that are related to the content of your manuscript. "Related" means any relation with for-profit or not-for-profit third parties whose interests may be affected by the content of the manuscript. Disclosure represents a commitment to transparency and does not necessarily indicate a bias. If you are in doubt about whether to list a relationship/activity/interest, it is preferable that you do so.

The following questions apply to the author's relationships/activities/interests as they relate to the <u>current manuscript only</u>.

The author's relationships/activities/interests should be <u>defined broadly</u>. For example, if your manuscript pertains to the epidemiology of hypertension, you should declare all relationships with manufacturers of antihypertensive medication, even if that medication is not mentioned in the manuscript.

In item #1 below, report all support for the work reported in this manuscript without time limit. For all other items, the time frame for disclosure is the past 36 months.

		**Name all entities with whom you have this relationship or indicate none (add rows as needed)**	**Specifications/Comments (e.g., if payments were made to you or to your institution)**
		**Time frame: Since the initial planning of the work**	
1	All support for the present manuscript (e.g., funding, provision of study materials, medical writing, article processing charges, etc.) **No time limit for this item.**	__ √ __None	
		**Time frame: past 36 months**	
2	Grants or contracts from any entity (if not indicated in item #1 above).	__ √ __None	
3	Royalties or licenses	__ √ __None	
4	Consulting fees	__ √ __None	

5	Payment or honoraria for lectures, presentations, speakers bureaus, manuscript writing or educational events	__ √ __None	
6	Payment for expert testimony	__ √ __None	
7	Support for attending meetings and/or travel	__ √ __None	
8	Patents planned, issued or pending	__ √ __None	
9	Participation on a Data Safety Monitoring Board or Advisory Board	__ √ __None	
10	Leadership or fiduciary role in other board, society, committee or advocacy group, paid or unpaid	__ √ __None	
11	Stock or stock options	__ √ __None	
12	Receipt of equipment, materials, drugs, medical writing, gifts or other services	__ √ __None	
13	Other financial or non-financial interests	__ √ __None	

Please place an "X" next to the following statement to indicate your agreement:

__X_ I certify that I have answered every question and have not altered the wording of any of the questions on this form.

ICMJE DISCLOSURE FORM

Date: ___ June-10th-2021____
Your Name: ___ Bo Zhang ____
Manuscript Title: ___ Ameliorating effects and mechanisms of transcutaneous auricular vagal nerve stimulation on abdominal pain and constipation ____
Manuscript number (if known): ___ 150052-INS-CMED-RV-3 ____

In the interest of transparency, we ask you to disclose all relationships/activities/interests listed below that are related to the content of your manuscript. "Related" means any relation with for-profit or not-for-profit third parties whose interests may be affected by the content of the manuscript. Disclosure represents a commitment to transparency and does not necessarily indicate a bias. If you are in doubt about whether to list a relationship/activity/interest, it is preferable that you do so.

The following questions apply to the author's relationships/activities/interests as they relate to the current manuscript only.

The author's relationships/activities/interests should be defined broadly. For example, if your manuscript pertains to the epidemiology of hypertension, you should declare all relationships with manufacturers of antihypertensive medication, even if that medication is not mentioned in the manuscript.

In item #1 below, report all support for the work reported in this manuscript without time limit. For all other items, the time frame for disclosure is the past 36 months.

		**Name all entities with whom you have this relationship or indicate none (add rows as needed)**	**Specifications/Comments (e.g., if payments were made to you or to your institution)**
**Time frame: Since the initial planning of the work**			
1	All support for the present manuscript (e.g., funding, provision of study materials, medical writing, article processing charges, etc.) **No time limit for this item.**	__ √ __None	
**Time frame: past 36 months**			
2	Grants or contracts from any entity (if not indicated in item #1 above).	__ √ __None	
3	Royalties or licenses	__ √ __None	
4	Consulting fees	__ √ __None	

5	Payment or honoraria for lectures, presentations, speakers bureaus, manuscript writing or educational events	__ √ __None	
6	Payment for expert testimony	__ √ __None	
7	Support for attending meetings and/or travel	__ √ __None	
8	Patents planned, issued or pending	__ √ __None	
9	Participation on a Data Safety Monitoring Board or Advisory Board	__ √ __None	
10	Leadership or fiduciary role in other board, society, committee or advocacy group, paid or unpaid	__ √ __None	
11	Stock or stock options	__ √ __None	
12	Receipt of equipment, materials, drugs, medical writing, gifts or other services	__ √ __None	
13	Other financial or non-financial interests	__ √ __None	

Please place an "X" next to the following statement to indicate your agreement:

__X_ I certify that I have answered every question and have not altered the wording of any of the questions on this form.

ICMJE DISCLOSURE FORM

Date: ___ June-10th-2021____
Your Name: ___ Wenna Li ____
Manuscript Title: ___ Ameliorating effects and mechanisms of transcutaneous auricular vagal nerve stimulation on abdominal pain and constipation ____
Manuscript number (if known): ___ 150052-INS-CMED-RV-3 ____

In the interest of transparency, we ask you to disclose all relationships/activities/interests listed below that are related to the content of your manuscript. "Related" means any relation with for-profit or not-for-profit third parties whose interests may be affected by the content of the manuscript. Disclosure represents a commitment to transparency and does not necessarily indicate a bias. If you are in doubt about whether to list a relationship/activity/interest, it is preferable that you do so.

The following questions apply to the author's relationships/activities/interests as they relate to the current manuscript only.

The author's relationships/activities/interests should be defined broadly. For example, if your manuscript pertains to the epidemiology of hypertension, you should declare all relationships with manufacturers of antihypertensive medication, even if that medication is not mentioned in the manuscript.

In item #1 below, report all support for the work reported in this manuscript without time limit. For all other items, the time frame for disclosure is the past 36 months.

		Name all entities with whom you have this relationship or indicate none (add rows as needed)	Specifications/Comments (e.g., if payments were made to you or to your institution)
		**Time frame: Since the initial planning of the work**	
1	All support for the present manuscript (e.g., funding, provision of study materials, medical writing, article processing charges, etc.) **No time limit for this item.**	__ √ __None	
		**Time frame: past 36 months**	
2	Grants or contracts from any entity (if not indicated in item #1 above).	__ √ __None	
3	Royalties or licenses	__ √ __None	
4	Consulting fees	__ √ __None	

5	Payment or honoraria for lectures, presentations, speakers bureaus, manuscript writing or educational events	__ √ __None	
6	Payment for expert testimony	__ √ __None	
7	Support for attending meetings and/or travel	__ √ __None	
8	Patents planned, issued or pending	__ √ __None	
9	Participation on a Data Safety Monitoring Board or Advisory Board	__ √ __None	
10	Leadership or fiduciary role in other board, society, committee or advocacy group, paid or unpaid	__ √ __None	
11	Stock or stock options	__ √ __None	
12	Receipt of equipment, materials, drugs, medical writing, gifts or other services	__ √ __None	
13	Other financial or non-financial interests	__ √ __None	

Please place an "X" next to the following statement to indicate your agreement:

__X_ I certify that I have answered every question and have not altered the wording of any of the questions on this form.

ICMJE DISCLOSURE FORM

Date: ___ June-10th-2021____
Your Name: ___ Jiande DZ Chen ____
Manuscript Title: ___ Ameliorating effects and mechanisms of transcutaneous auricular vagal nerve stimulation on abdominal pain and constipation ____
Manuscript number (if known): ___ 150052-INS-CMED-RV-3 ____

In the interest of transparency, we ask you to disclose all relationships/activities/interests listed below that are related to the content of your manuscript. "Related" means any relation with for-profit or not-for-profit third parties whose interests may be affected by the content of the manuscript. Disclosure represents a commitment to transparency and does not necessarily indicate a bias. If you are in doubt about whether to list a relationship/activity/interest, it is preferable that you do so.

The following questions apply to the author's relationships/activities/interests as they relate to the current manuscript only.

The author's relationships/activities/interests should be defined broadly. For example, if your manuscript pertains to the epidemiology of hypertension, you should declare all relationships with manufacturers of antihypertensive medication, even if that medication is not mentioned in the manuscript.

In item #1 below, report all support for the work reported in this manuscript without time limit. For all other items, the time frame for disclosure is the past 36 months.

		**Name all entities with whom you have this relationship or indicate none (add rows as needed)**	**Specifications/Comments (e.g., if payments were made to you or to your institution)**
		**Time frame: Since the initial planning of the work**	
1	All support for the present manuscript (e.g., funding, provision of study materials, medical writing, article processing charges, etc.) **No time limit for this item.**	__ √ __None	
		**Time frame: past 36 months**	
2	Grants or contracts from any entity (if not indicated in item #1 above).	__ √ __None	
3	Royalties or licenses	__ √ __None	
4	Consulting fees	__ √ __None	

5	Payment or honoraria for lectures, presentations, speakers bureaus, manuscript writing or educational events	__ √ __None	
6	Payment for expert testimony	__ √ __None	
7	Support for attending meetings and/or travel	__ √ __None	
8	Patents planned, issued or pending	__ √ __None	
9	Participation on a Data Safety Monitoring Board or Advisory Board	__ √ __None	
10	Leadership or fiduciary role in other board, society, committee or advocacy group, paid or unpaid	__ √ __None	
11	Stock or stock options	__ √ __None	
12	Receipt of equipment, materials, drugs, medical writing, gifts or other services	__ √ __None	
13	Other financial or non-financial interests	__ √ __None	

Please place an "X" next to the following statement to indicate your agreement:

__X_ I certify that I have answered every question and have not altered the wording of any of the questions on this form.

ICMJE DISCLOSURE FORM

Date: ___ June-10th-2021____
Your Name: ___ Fei Liu____
Manuscript Title: ___ Ameliorating effects and mechanisms of transcutaneous auricular vagal nerve stimulation on abdominal pain and constipation ______________________________________
Manuscript number (if known): ___ 150052-INS-CMED-RV-3 _____________________________

In the interest of transparency, we ask you to disclose all relationships/activities/interests listed below that are related to the content of your manuscript. "Related" means any relation with for-profit or not-for-profit third parties whose interests may be affected by the content of the manuscript. Disclosure represents a commitment to transparency and does not necessarily indicate a bias. If you are in doubt about whether to list a relationship/activity/interest, it is preferable that you do so.

The following questions apply to the author's relationships/activities/interests as they relate to the <u>current manuscript only</u>.

The author's relationships/activities/interests should be <u>defined broadly</u>. For example, if your manuscript pertains to the epidemiology of hypertension, you should declare all relationships with manufacturers of antihypertensive medication, even if that medication is not mentioned in the manuscript.

In item #1 below, report all support for the work reported in this manuscript without time limit. For all other items, the time frame for disclosure is the past 36 months.

		**Name all entities with whom you have this relationship or indicate none (add rows as needed)**	**Specifications/Comments (e.g., if payments were made to you or to your institution)**
		**Time frame: Since the initial planning of the work**	
1	All support for the present manuscript (e.g., funding, provision of study materials, medical writing, article processing charges, etc.) **No time limit for this item.**	__ √ __None	
		**Time frame: past 36 months**	
2	Grants or contracts from any entity (if not indicated in item #1 above).	__ √ __None	
3	Royalties or licenses	__ √ __None	
4	Consulting fees	__ √ __None	

5	Payment or honoraria for lectures, presentations, speakers bureaus, manuscript writing or educational events	__ √ __None	
6	Payment for expert testimony	__ √ __None	
7	Support for attending meetings and/or travel	__ √ __None	
8	Patents planned, issued or pending	__ √ __None	
9	Participation on a Data Safety Monitoring Board or Advisory Board	__ √ __None	
10	Leadership or fiduciary role in other board, society, committee or advocacy group, paid or unpaid	__ √ __None	
11	Stock or stock options	__ √ __None	
12	Receipt of equipment, materials, drugs, medical writing, gifts or other services	__ √ __None	
13	Other financial or non-financial interests	__ √ __None	

Please place an "X" next to the following statement to indicate your agreement:

__X_ I certify that I have answered every question and have not altered the wording of any of the questions on this form.
